# Association between Common Variants near *LBX1* and Adolescent Idiopathic Scoliosis Replicated in the Chinese Han Population

**DOI:** 10.1371/journal.pone.0053234

**Published:** 2013-01-04

**Authors:** Wenjie Gao, Yan Peng, Guoyan Liang, Anjing Liang, Wei Ye, Liangming Zhang, Swarkar Sharma, Peiqiang Su, Dongsheng Huang

**Affiliations:** 1 Department of Orthopedic Surgery, The First Affiliated Hospital of Sun Yat-sen University, Guangdong, China; 2 Department of Orthopedic Surgery, Sun Yat-sen Memorial Hospital of Sun Yat-sen University, Guangzhou, Guangdong, China; 3 Seay Center for Musculoskeletal Research, Texas Scottish Rite Hospital for Children, Dallas, Texas, United States of America; 4 School of Biology and Chemistry, Shri Mata Vaishno Devi University, Katra, India; University of Bristol, United Kingdom

## Abstract

**Background:**

Adolescent idiopathic scoliosis (AIS) is one of the most common spinal deformities found in adolescent populations. Recently, a genome-wide association study (GWAS) in a Japanese population indicated that three single nucleotide polymorphisms (SNPs), rs11190870, rs625039 and rs11598564, all located near the *LBX1* gene, may be associated with AIS susceptibility [1]. This study suggests a novel AIS predisposition candidate gene and supports the hypothesis that somatosensory functional disorders could contribute to the pathogenesis of AIS. These findings warrant replication in other populations.

**Methodology/Principal Findings:**

First, we conducted a case-control study consisting of 953 Chinese Han individuals from southern China (513 patients and 440 healthy controls), and the three SNPs were all found to be associated with AIS predisposition. The ORs were observed as 1.49 (95% CI 1.23–1.80, *P* = 5.09E-5), 1.70 (95% CI 1.42–2.04, *P* = 1.17E-8) and 1.52 (95% CI 1.27–1.83, *P* = 5.54E-6) for rs625039, rs11190870 and rs11598564, respectively. Second, a case-only study including a subgroup of AIS patients (N = 234) was performed to determine the effects of these variants on the severity of the condition. However, we did not find any association between these variants and the severity of curvature.

**Conclusion:**

This study shows that the genetic variants near the *LBX1* gene are associated with AIS susceptibility in Chinese Han population. It successfully replicates the results of the GWAS, which was performed in a Japanese population.

## Introduction

Adolescent idiopathic scoliosis (AIS) is a structural, tridimensional spinal deformity characterized by lateral curvature of the spine with Cobb angle (which is a measurement used for evaluation of curves in scoliosis [2]) greater than 10°. It affects 2–3% of the adolescent populations [3].

Heritable and genetic factors have been found to play a vital role in the occurrence and development of AIS [4], [5]. Several loci associated with predisposition to AIS have been identified in genome-wide linkage studies in such regions as 6p, 10q, 18q, 19p13.3, 17p11, 19p13, 8q12, 9q31.2-q34.2, 17q25.3-qtel, 12p, and Xq [6]–[14]. Single nucleotide polymorphisms (SNPs) in the genes for estrogen receptor α (*ESR1*), estrogen receptor β (*ESR2*), matrilin 1 (*MATN1*), melatonin receptor 1B (*MTNR1B*), tryptophan hydroxylase 1(*TPH1*), interleukin-6 (*IL-6*) and matrix metalloproteinase-3 (*MMP-3*) have been reported to be associated with AIS predisposition [15]–[20]. However, so far these studies have not been replicated in other ethnic groups [21]–[25]. Polymorphisms of *ESR1, ESR2, MATN1*, insulin-like growth factor-I (*IGF-I*), tissue inhibitor of metalloproteinase-2 (*TIMP-2*), G protein-coupled estrogen receptor 1 (*GPER*), and neurotrophin 3 (*NTF3*) have been reported to be associated with the severity of curvature in AIS [15]–[17], [26]–[30]. These might be the modifier genes for AIS, but at present there is a lack of conclusive functional studies [4].

Genetic association studies are the means of identifying risk variants in complex traits, and replication studies that confirm their findings in other ethnic groups are quite necessary [4], [31]–[33]. Recently, a genome-wide association study (GWAS) was performed in a Japanese population, and three SNPs (rs11190870, rs625039 and rs11598564), all of which were located near the gene *LBX1* on chromosome 10q24.31, were reported to be associated with AIS susceptibility [1]. Two association studies conducted in Chinese Han population from Hong Kong and Yangtze River region of mainland China replicated the association between AIS and rs1190870 [34], [35], and rs11598564 was among the top 100 SNPs identified in a GWAS conducted in the United States [36].

In order to determine whether rs11190870, rs625039, and rs11598564 are associated with a predisposition to AIS in Chinese Han population of Southern China, we conducted a case-control study involving 513 AIS patients and 440 control subjects. A case-only study including a subgroup of AIS patients was also performed to determine the effects of these variants on the severity of the condition.

## Methods

### Ethics Statement

The study has been approved by the Ethical Committee of the First Affiliated Hospital of Sun Yat-sen University and the Ethical Committee of Sun Yat-sen Memorial Hospital of Sun Yat-sen University. Written informed consent was obtained from all subjects or their parents in the case of children.

### Study Participants

All the individuals who participated in the present study were of Chinese Han ethnicity and from Guangdong Province in Southern China.

Five hundred and thirteen AIS patients were collected from the First Affiliated Hospital and Sun Yat-sen Memorial Hospital of Sun Yat-sen University. They included 336 patients with mild scoliosis (who only needed observation), 108 with moderate scoliosis (treated with bracing), and 69 with severe scoliosis (treated with surgery). The diagnosis of idiopathic scoliosis is one of exclusion, and it is made only when other causes of scoliosis have been ruled out [37], [38]. Based on the age of the patient at the time of his or her first diagnosis of scoliosis, idiopathic scoliosis can be subdivided into three groups: infantile, before three years of age; juvenile, between three and ten years of age; and adolescent, between age ten and skeletal maturity [37], [39]. In our study, all patients provided detailed histories, accepted physical examinations, underwent standard up-standing posteroanterior radiography of the whole spine, and other testing (if necessary), such as MRI, CT, and nuclear scintigraphy etc [37], [40]). All patients were ascertained for diagnosis of AIS at the age of 10–16 years by at least two spinal surgeons. Patients with congenital scoliosis and scoliosis secondary to neuromuscular disorders, endocrine disorders, skeletal dysplasia, connective tissue abnormalities, and syndromic disorders were excluded. The controls typed for the analysis comprised: 1) 363 young students recruited during scoliosis screening at middle and primary schools; 2) 77 young fracture patients selected from the First Affiliated Hospital and Sun Yat-sen Memorial Hospital of Sun Yat-sen University. Adam’s forward bend test and scoliometers were used to screen individuals for scoliosis [37], [38]. (The sensitivities of the Adam’s forward bend test and scoliometer have been reported to be 84.37% and 90.62%, respectively, and specificity is 93.44% and 79.76% respectively [41].) Radiographs were taken for validation in case of any uncertainty. Routine history-taking and physical examinations were also conducted to exclude other deformities of the skeletal system, hereditary diseases, and disorders affecting bone growth and metabolism. At least two orthopedic surgeons assessed the controls. Only when all surgeons were in agreement was the participant included in the study.

AIS tends to progress until skeletal maturity, and bracing can change the natural course of the condition [42]. Only a subgroup of AIS patients (N = 234), those who reached the endpoints of curve progression [surgical fusion for AIS or skeletal maturity (defined as age 16 or Risser sign 5)] and had not undergone bracing, were included in the case-only study. The severity of spinal curvature was measured using the Cobb method on standard up-standing posteroanterior radiography of the whole spine [2]. The measurement was made by drawing tangents along the superior endplate of the superior end vertebra and the inferior endplate of the inferior end vertebra. The Cobb angle was here defined as either the angle between the tangential lines or the angle between two lines drawn perpendicular to the tangents (the two angles are identical) [43]. The maximum Cobb angle (MCA) of the primary curve was used to assess the severity of AIS. For patients who underwent surgery, MCA was taken before surgery, and for those who were under observation, MCA was taken during the latest follow-up.

After written informed consent was obtained, participants’ basic information, such as name, age, sex, ethnic group, and birthplace, and clinical information, such as Cobb angle and Risser sign [44], [45], were recorded, and blood samples were collected.

### Genotyping

Genomic DNA was isolated from 200 µl blood per patient using Tiangen DNA Blood Mini Kits (Tiangen, Beijing, China) according to the manufacturer’s instructions. Genomic DNA was diluted to a final concentration of 10–15 ng/µL for genotyping assays. Polymorphism-spanning fragments were amplified using polymerase chain reaction (PCR) and genotyped using the MassArray system (Sequenom, San Diego, CA, U.S.) with primers (Table S1) at the Beijing Genomics Institute in Shenzhen, China, as described previously [46]. For quality control, three template-free controls and sixteen duplicated samples were used per 384-sample plate, and the results were 100% consistent. Genotyping was conducted blindly, and the call rate for each SNP was 100%. The genotype distributions of the SNPs were all in Hardy-Weinberg equilibrium.

### Statistical Analysis

Hardy–Weinberg equilibrium (HWE) was evaluated in both the case and control groups for all SNPs. Differences between cases and controls with respect to allele frequency were evaluated using the Chi-Square test, and the Cochran-Armitage trend test was used for genotype frequency. The allelic odds ratios (ORs) and their 95% confidence interval (CI) ranges were calculated. Bonferroni adjustment was performed for multiple-test corrections. Logistic regression was used to adjust the confounding effects of age and sex and determine the effect of interaction between SNP and sex. The Kruskal–Wallis test was used to evaluate the association between age and genotype. The Kruskal–Wallis test and ordinary least squared regression were used in the comparison of maximum Cobb angles (MCAs) among different genotypes. Linear regression based beta coefficients were also calculated to reflect the changes in MCAs per risk allele. The power calculation for the association study was performed with the NCSS/PASS software (NCSS, Kaysville, UT, USA). Linkage disequilibrium (LD) and haplotype analyses were performed using Haploview 4.2 [47]. The other statistical tests were performed using SPSS 13.0 (SPSS Inc., Chicago, IL, U.S.) and PLINK 1.07 [48].

## Results

### Case–control Study

This study included 513 AIS patients with Cobb angles over 15° and 440 control subjects (Table 1). The distributions of the alleles and genotypes for the three SNPs are given in Table 2. All the SNPs were all found to be associated with AIS predisposition, the ORs were observed as 1.49 (95% CI 1.23–1.80, *P* = 5.09E-5), 1.70 (95% CI 1.42–2.04, *P* = 1.17E-8) and 1.52 (95% CI 1.27–1.83, *P* = 5.54E-6) for rs625039, rs11190870 and rs11598564, respectively. And the present study had a power of 91%, 91% and 77% for rs625039, rs11190870 and rs11598564 respectively to show the effects reported in the previous GWAS [1].

**Table 1:** Characteristics of the study population.

Variables	Cases	Controls
Ethnic group	Chinese Han	Chinese Han
Birthplace	Guangdong Province	Guangdong Province
N (observe/bracing/surgery)	513(336/108/69)	440(NA^c^)
Female/male	447/66	289/151
Mean age±SD^a^ (years)	15.82±3.32	14.46±2.22
Age range (years)	10–30	8–25
Age at diagnosis (years)	10–16	NA
Mean MCA^b^±SD (°)	25.57±14.10	NA
MCA range (°)	15–140	NA

a Standard deviation (SD).

b The maximum Cobb angle (MCA).

c Not applicable (NA).

**Table 2:** Association between the SNPs near *LBX1* and AIS predisposition in Chinese Han population.

			Major/minor allele	Case					Control						
				Genotype count					Genotype count				OR^c^	*P* (*P* e *_adust_*)	
SNP	Position	Location		11	12	22	RAF^b^		11	12	22	RAF	(95% CI d)	Genotype^f^	Allele^g^
rs625039	53798113	5′-flanking	**G** a/A	257	218	38	0.714		176	199	65	0.626	1.49	5.35E-5	5.09E-5
		region											(1.23–1.80)	(1.61E-4)	(1.53E-4)
rs11190870	53783671	3′-flanking	**T**/C	200	236	77	0.620		114	203	123	0.490	1.70	3.26E-8	1.17E-8
		region											(1.42–2.04)	(9.78E-8)	(3.51E-8)
rs11598564	53769068	3′-flanking	**G**/A	185	246	82	0.600		115	207	118	0.497	1.52	8.28E-6	5.54E-6
		region											(1.27–1.83)	(2.48E-5)	(1.66E-5)

a Risk alleles were indicated in boldface.

b Risk allele frequency (RAF).

c Allelic odds ratio.

d Confidence interval (CI).

e *P*-values were adjusted using the Bonferroni method for multiple tests.

f *P* -values were calculated using the Cochran-Armitage trend test.

g *P* -values were calculated using the χ^2^ test.

The linkage disequilibrium coefficient (r^2^) between rs625039 and rs11190870 was 0.59, and the coefficient between rs11190870 and rs11598564 was 0.70 (Figure S1). Haplotype analysis did not show any association stronger than that observed between the SNP rs11190870 and AIS (Table 3).

**Table 3:** Haplotype configurations among the three SNPs.

rs11598564	rs11190870	rs625039	Case frequency	Control frequency	Haplotype	*P*
**G** a	**T**	**G**	0.569	0.449	Hap 1	1.72E-7
A	C	A	0.268	0.350	Hap 2	1.00E-4
A	C	**G**	0.083	0.116	Hap 3	0.016
A	**T**	**G**	0.047	0.037	Hap 4	0.26
**G**	C	**G**	0.014	0.024	Hap 5	0.10
**G**	C	A	0.016	0.020	Hap 6	0.44

a Risk alleles were indicated in boldface.

We also evaluated possible confounding factors, such as age and sex using logistic regression, and the adjusted ORs for rs625039, rs11190870 and rs11598564 were 1.60 (95% CI 1.26–2.03), 1.65 (95% CI 1.36–2.01), and 1.51(95% CI 1.24–1.84), respectively (Table S2). No interaction effect between SNP and sex on the risk of AIS was observed (Table S2). Also, no association was found between age and genotype for the SNPs in either the case or control group (Table 4). When the population was stratified by sex, associations between SNPs and AIS predisposition were detected among female individuals, but not among male individuals. However, this might be due to the small sample size of male subset (66 cases and 151 controls) (Table 5).

**Table 4:** Association between age and genotype.

		Mean±SD age (years) for genotype			
SNP	Group	11	12	22	*P* a
rs625039	Control	14.33±1.98	14.42±2.66	14.61±2.02	0.96
	Case	16.06±4.32	15.50±2.89	16.39±4.45	0.58
rs11190870	Control	14.43±1.98	14.44±2.66	14.37±1.97	1.00
	Case	16.09±4.40	15.71±3.25	15.65±3.65	0.87
rs11598564	Control	14.39±1.93	14.47±2.66	14.33±1.99	0.87
	Case	16.19±4.54	15.65±3.18	15.67±3.64	0.55

a *P*-values were calculated using Kruskal–Wallis test.

**Table 5:** Association between the SNPs and AIS predisposition, stratified by sex.

		Case					Control							
		Genotype count					Genotype count				OR^b^	*P* (*P* d *_adust_*)		
SNP	Sex	11	12	22	RAF^a^		11	12	22	RAF	(95% CI c)	Genotype^e^		Allele^f^
rs625039	Male	36	23	7	0.720		59	70	22	0.623	1.56	0.057		0.051
											(1.00–2.43)	(0.17)		(0.15)
	Female	221	195	31	0.713		117	129	43	0.628	1.47	6.50E-4		6.94E-4
											(1.18–1.83)	(1.95E-3)		(2.08E-3)
rs11190870	Male	29	23	14	0.614		40	69	42	0.493	1.63	0.031		0.021
											(1.08–2.47)	(0.093)		(0.063)
	Female	171	213	63	0.621		74	134	81	0.488	1.72	8.10E-7		4.95E-7
											(1.39–2.12)	(2.43E-6)		(1.49E-6)
rs11598564	Male	26	27	13	0.599		44	65	42	0.507	1.45	0.099		0.078
											(0.96–2.20)	(0.30)		(0.23)
	Female	159	219	69	0.601		71	142	76	0.491	1.56	3.92E-5		3.72E-5
											(1.26–1.92)	(1.18E-4)		(1.12E-4)

a Risk allele frequency (RAF).

b Allelic odds ratio.

c Confidence interval (CI).

d *P*-values were adjusted using the Bonferroni method for multiple tests.

e *P* -values were calculated using the Cochran-Armitage trend test.

f *P* -values were calculated using the χ^2^ test.

### Case-only Study

This study included 234 AIS patients who had reached the scoliosis curve endpoints [surgical fusion for AIS or skeletal maturity (defined as age 16 or Risser sign 5)] and had never been braced. No difference with respect to MCAs was found among genotypes for any of the SNPs (Table 6). A sensitivity analysis was also performed on all 234 cases and 440 controls. The SNPs were still found to be associated with AIS predisposition, and the ORs showed little difference from the previous case-control study (Table S3). In this way, in the present study, none of the SNPs were found to be associated with the severity of spinal curvature.

**Table 6:** Association between SNPs and the severity of spinal curvature in AIS.

SNP	Genotype	Number	Mean MCA^a^ ± SD(°)	β coefficient (Standard error) b	*P* b (*P* c)
rs625039	AA	17	31.35±22.07	1.03 (1.82)	0.57 (0.37)
	AG	95	28.73±17.89		
	GG	122	31.00±16.68		
rs11190870	CC	31	27.90±18.05	0.65 (1.68)	0.70 (0.33)
	TC	110	30.73±19.56		
	TT	93	30.10±14.81		
rs11598564	AA	34	28.32±17.26	0.66 (1.66)	0.69(0.50)
	GA	110	30.60±19.58		
	GG	90	30.18±15.02		

a Maximum Cobb angle (MCA).

b β coefficients, standard errors and *P*-values were calculated using ordinary least squared regression.

c *P*-values were calculated using the Kruskal–Wallis test.

## Discussion

In the case-control study, all three genotyped SNPs were found to be associated with AIS predisposition. We also found G, T, and G to be risk alleles for rs11598564, rs11190870, and rs625039, respectively, the same as those reported in the previous Japanese GWAS [1]. The association between common variants near *LBX1* and AIS predisposition found in Japanese population was successfully replicated in this Chinese Han population.

Studies on animal models showed that damage to the sensory area of the spinal cord or posterior rhizotomy could cause scoliosis, indicating that somatosensory dysfunction might play a significant role in AIS [49]–[52]. In clinical studies, it has been noted that growing children with functional or structural disorders of the somatosensory pathway are more susceptible to scoliosis than their healthy counterparts [5]. In AIS patients, the prevalence of somatosensory disorders is much higher than in the general population [53]–[55]. *LBX1* is a homeobox gene expressed in the dorsal part of the spinal cord and hindbrain. It was first cloned by Jagla K et al., and it has been reported to act as a selector gene in the determination of the fates of dorsal spinal and hindbrain somatosensory neurons [56]–[61]. In *Lbx1−/−* mice, the morphology and neuronal circuitry of the dorsal horn are aberrant, suggesting that *LBX1* is critical to the development of the sensory pathway in the spinal cord [58]. Because variants near *LBX1* were found to be associated with predisposition to AIS in the present and previous studies, it is possible that abnormal *LBX1* expression might contribute to AIS by causing somatosensory function disorders [1].

Replication is essential for substantiation of the positive findings of association studies and identification of common causes of disease among different populations. However, this process often fails in independent studies, including AIS association studies [21]–[25]. Since Xu S etc. [62] reported that there are remarkable genetic differences among Chinese Han populations from different regions of China, ours and other recent two association studies [34], [35], which were conducted in Chinese Han population from different regions of China (from Hong Kong, Yangtze River region and Southern region of China respectively), all replicated the association between AIS and rs1190870, and strongly support that rs11190870 may account for disease predisposition of AIS in Chinese Han population. Yet further replication studies must be performed in other ethnic groups, since of the SNPs evaluated here, only rs11598564 was among the top 100 SNPs identified in a recent large-scale GWAS conducted in the United States [36]. It is also important to note that these three SNPs are all located in the flanking region of *LBX1* (rs11190870 and rs11598564 are in the 3′ region, and rs625039 is in the 5′ region). The potential functions of these SNPs are still unclear. These variants may act as regulatory elements for *LBX1*, affecting the quality and quantity of *LBXI* mRNA [63], [64]. Then we searched these SNPs in the recently published ENCODE database (http://www.regulomedb.org), and observed rs625039 may minimally affect binding of motif Pax-4; rs11190870 may minimally affect binding of 9 motifs (Lhx5, Pbx-1b, Oct-4, Oct-1, Pou2f2, Lhx3, Pou2f3, Arid3a and Octamer), whereas, rs11598564 may not cause any motif change. It is also possible that these are just markers of LD, associated with actual disease-causing variants. Functional analysis of these variants and targeted resequencing of the whole LD block must be performed to identify functional variants. This may shed further light on the mechanisms underlying the pathogenesis of AIS.

Adjusting the association for age and sex effects using a logistic regression approach did not substantially change the nature of our findings. The adjusted ORs showed little difference from the pre-adjustment values (Table S2). No association was found between age and genotype for any SNP, indicating that age has no effect on the distribution of the genotypes evaluated in the present study. Although the control group included subjects who were young and had immature skeletal systems [(<10 years) 7; (≥10 and <14 years) 135; (≥14 and <16 years) 223; (>16 years) 75] at the time of sample collection, some of them might go on to develop the disease over time. However, in light of a previous epidemiological studies, only a very small number of them (<1%) would develop clinically significant AIS [3].

Disease modifier genes might be useful for predicting the progression of disease and helpful in early clinical investigation and treatment [4]. For this reason, we conducted a case-only study including a subgroup of AIS patients. Because AIS can progress until skeletal maturity and bracing can change the natural course of AIS, only patients who had reached the endpoints of curve progression without ever having been braced were included [42]. In the present study, we found none of these three SNPs to be associated with the severity of spinal curvature in AIS, which indicates that *LBX1* might not be a disease modifier gene for AIS. However, further study with larger sample size and conclusive functional study are needed to confirm this.

This study has some limitations that should be addressed. First, a significant difference was observed between male and female individuals with respect to prevalence of AIS. The ratio of female-to-male prevalence was found to be 3.6∶1 [3]. When we stratified the sample by sex, little evidence for association was found among males (Table 5). Because the variants were found to have the same effects on AIS predisposition in female individuals from both Chinese and Japanese populations, and no interaction effect between SNP and sex was observed (Table S2), we conclude that a lack of power [due to the small size of the male sample population (66 cases and151 controls)], rather than the sex specific effects of the variants in Chinese Han population, is the most plausible explanation for this lack of association. Further study with larger sample size is needed. Second, AIS is a complex trait, and certain non-genetic risk factors for AIS, such as oteopenia and late menarche have been reported [4], [65], [66]. However, these data were not collected in the present study, which limited our ability to evaluate of gene-environment interactions.

## Supporting Information

Figure S1 **Linkage disequilibrium among the SNPs was measured using (a) D′ and (b) r^2^.**
(JPG)

Table S1 **Primer sequences used for genotyping the SNPs with the Sequenom platform.**
(DOC)

Table S2 **Adjustment for age and sex and investigation of the interaction effect between SNP and sex using logistic regression.**
(DOC)

Table S3 **Case-control study with 234 cases in whom the severity of the curve was measurable and 440 controls.**
(DOC)
