# Supplementary figures and images for: Association between Common Variants near LBX1 and Adolescent Idiopathic Scoliosis Replicated in the Chinese Han Population
Source: PLoS One. 2013 Jan 4;8(1):e53234. doi: 10.1371/journal.pone.0053234 (PMC3537668; doi:10.1371/journal.pone.0053234)

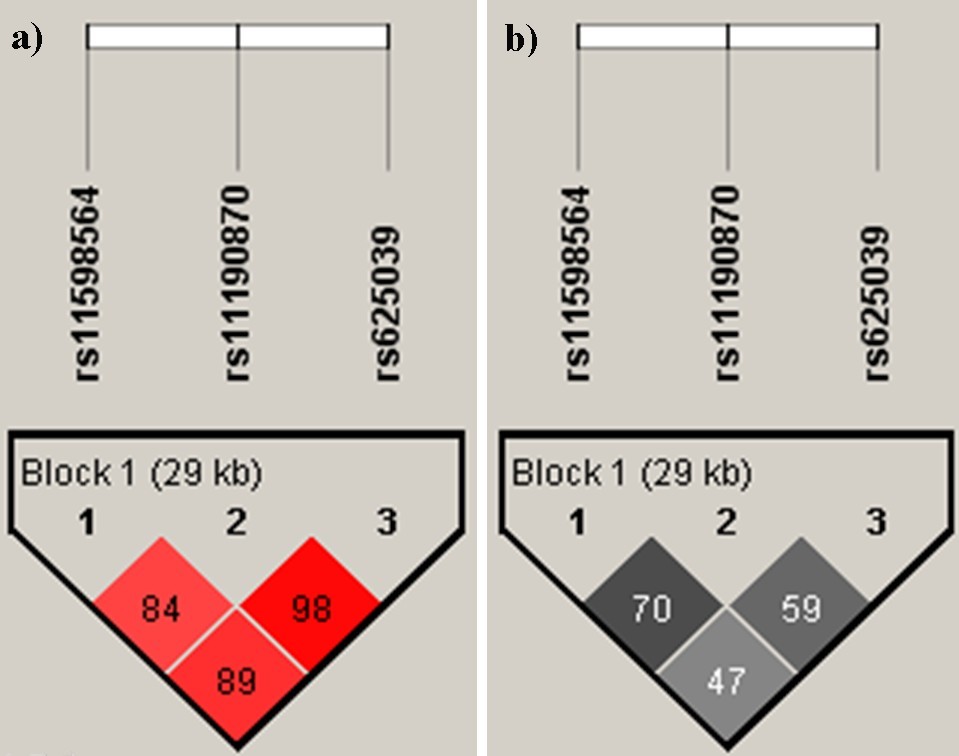

Supplement: Figure S1 — Linkage disequilibrium among the SNPs was measured using (a) D′ and (b) r2. (JPG) [file pone.0053234.s001.jpg]
