# Supplementary material for: Association between Common Variants near LBX1 and Adolescent Idiopathic Scoliosis Replicated in the Chinese Han Population
Source: PLoS One. 2013 Jan 4;8(1):e53234. doi: 10.1371/journal.pone.0053234 (PMC3537668; doi:10.1371/journal.pone.0053234)
Supplement: Table S1 — Primer sequences used for genotyping the SNPs with the Sequenom platform. (DOC) [file pone.0053234.s002.doc]

**Table S1. Primer sequences used for genotyping the SNPs with the Sequenom platform**

| **SNP** | **Forward primers** | **Reverse primers** | **Extension primers** |
| --- | --- | --- | --- |
| rs625039 | ACGTTGGATGAACACCCAGGATTCTCAAAG | ACGTTGGATGTCCCTACAGTGGCCTGATTC | GATTCTCAAAGCGGGTG |
| rs11190870 | ACGTTGGATGCCAACACCAGAGGAATTATC | ACGTTGGATGTATGGAGCTGTTTGCCTGCG | GGAATTATCAACTAGAATTTGATTAATA |
| rs11598564 | ACGTTGGATGCCTGTTGAGTCAGTATCCAC | ACGTTGGATGCGCCGAACTTCTCCAACTTC | TTTTCCTGAGAGCTTCTTG |
