# Supplementary material for: Association between Common Variants near LBX1 and Adolescent Idiopathic Scoliosis Replicated in the Chinese Han Population
Source: PLoS One. 2013 Jan 4;8(1):e53234. doi: 10.1371/journal.pone.0053234 (PMC3537668; doi:10.1371/journal.pone.0053234)
Supplement: Table S2 — Adjustment for age and sex and investigation of the interaction effect between SNP and sex using logistic regression. (DOC) [file pone.0053234.s003.doc]

**Table S2.** Adjustment for age and sex and investigation of the interaction effect between SNP and sex using logistic regression

|  |  | ***P*** | |
| --- | --- | --- | --- |
| **SNP** | **OR^a^ (95%CI^b^)** | **Genotype^c^** | **SNP × Sex^d^** |
| rs625039 | 1.60 (1.26–2.03) | 1.01E-4 | 0.95 |
| rs11190870 | 1.65 (1.36–2.01) | 4.44E-7 | 0.42 |
| rs11598564 | 1.51 (1.24–1.84) | 3.71E-5 | 0.36 |

^a^ odds ratio

^b^ Confidence interval (CI)

^c^*P*-values for the effect of the SNP after adjusting age and sex.

^d^*P*-values for the interaction effect of SNP and sex.
