# Supplementary material for: Association between Common Variants near LBX1 and Adolescent Idiopathic Scoliosis Replicated in the Chinese Han Population
Source: PLoS One. 2013 Jan 4;8(1):e53234. doi: 10.1371/journal.pone.0053234 (PMC3537668; doi:10.1371/journal.pone.0053234)
Supplement: Table S3 — Case-control study with 234 cases in whom the severity of the curve was measurable and 440 controls. (DOC) [file pone.0053234.s004.doc]

**Table S3.** Case-control study with 234 cases in whom the severity of the curve was measurable and 440 controls

|  | **Genotype count** | |  |  |  | |  |
| --- | --- | --- | --- | --- | --- | --- | --- |
|  | **(11/12/22)** | | **RAF^a^** | **OR^b^** | ***P*(*P* ^d^*_adust_*)** | | |
| **SNP** | **Case** | **Control** | **(case/control)** | **(95% CI ^c^)** | **Genotype^e^** | **Allele^f^** | |
| rs625039 | 122/95/17 | 176/199/65 | 0.724/0.626 | 1.57 | 3.49E-4 | 2.89E-4 | |
|  |  |  |  | (1.23–2.00) | (1. 05E-3) | (8.67E-4) | |
| rs11190870 | 93/110/31 | 114/203/123 | 0.633/0.490 | 1.79 | 1.25E-6 | 5.61E-7 | |
|  |  |  |  | (1.43–2.26) | (3.75E-6) | (1.68E-6) | |
| rs11598564 | 90/110/34 | 115/207/118 | 0.620/0.497 | 1.65 | 2.62E-5 | 1.59E-5 | |
|  |  |  |  | (1.31–2.08) | (7.86E-5) | (4.77E-5) | |

**^a^**Risk allele frequency (RAF).

**^b^**Allelic odds ratio.

**^c^**Confidence interval (CI).

**^d^***P*-values were adjusted using the Bonferroni method for multiple tests.

**^e^***P* -values were calculated using the Cochran-Armitage trend test.

**^f^***P* -values were calculated using the χ^2^.
